# Transcriptional Activation of Biosynthetic Gene Clusters in Filamentous Fungi

**DOI:** 10.3389/fbioe.2022.901037

**Published:** 2022-07-15

**Authors:** László Mózsik, Riccardo Iacovelli, Roel A. L. Bovenberg, Arnold J. M. Driessen

**Affiliations:** ^1^ Department of Molecular Microbiology, Groningen Biomolecular Sciences and Biotechnology Institute, University of Groningen, Groningen, Netherlands; ^2^ DSM Biotechnology Center, Delft, Netherlands; ^3^ Department of Synthetic Biology and Cell Engineering, Groningen Biomolecular Sciences and Biotechnology Institute, University of Groningen, Groningen, Netherlands

**Keywords:** secondary metabilites, biosynthetic gene cluster, synthetic biology, synthetic transcriptional regulators, fungal platform strains

## Abstract

Filamentous fungi are highly productive cell factories, many of which are industrial producers of enzymes, organic acids, and secondary metabolites. The increasing number of sequenced fungal genomes revealed a vast and unexplored biosynthetic potential in the form of transcriptionally silent secondary metabolite biosynthetic gene clusters (BGCs). Various strategies have been carried out to explore and mine this untapped source of bioactive molecules, and with the advent of synthetic biology, novel applications, and tools have been developed for filamentous fungi. Here we summarize approaches aiming for the expression of endogenous or exogenous natural product BGCs, including synthetic transcription factors, assembly of artificial transcription units, gene cluster refactoring, fungal shuttle vectors, and platform strains.

## Introduction

Secondary metabolites (SM), commonly referred to as natural products, are chemical substances that are produced by living organisms, often bearing distinctive pharmacological effects. The exploitation of microorganisms for generating these valuable products for our societies in an economical manner has a great history. Notably, the use of filamentous fungi in industrial biotechnology is well established. With the introduction of synthetic biology, new tools and alternative methods are provided to further aid the metabolic engineering and exploitation of fungal workhorses. Filamentous fungi (with key players from *Aspergillus*, *Penicillium*, *Trichoderma*, *Fusarium*, and *Neurospora* species) are highly efficient cell factories, often used industrially for the production of a diverse range of products such as proteins, enzymes, organic acids, and SMs (Meyer et al., 2020). SMs are not essential for the survival of the organism, but the production of these natural products often provides an evolutionary advantage. With the discovery of penicillin in 1928 produced by a mold identified as a *Penicillium* species, a new era started for industrial antibiotics production and the exploration and characterization of novel fungal SMs. This interest resulted in the discovery of not just antibacterial, but also antifungal (griseofulvin), cholesterol-lowering (lovastatin), immunosuppressant (cyclosporine), anticancer (paclitaxel), and food additive (carmine) compounds (Keller et al., 2005). Alongside the beneficial metabolites, fungi also produce SMs acting as toxins (e.g., aflatoxin, fumonisin, patulin), which negatively affect food, feed, livestock, and human health.

**FIGURE 1:**
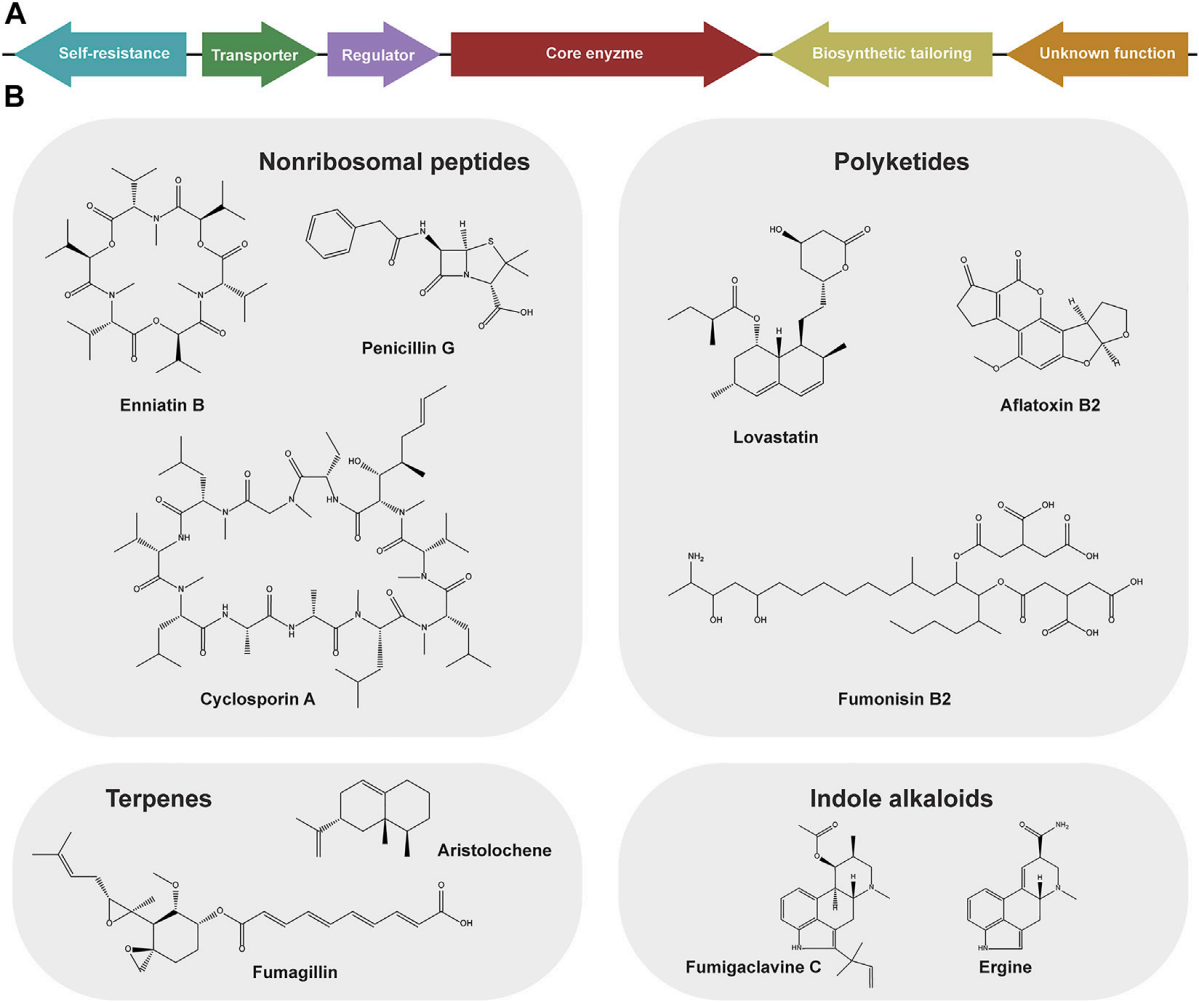
Schematic representation of a fungal biosynthetic gene cluster (BGCs) **(A)** and structurally different, representative members of nonribosomal peptides synthetase (NRPS), and polyketides synthase (PKS), terpenes synthase produced secondary metabolites and indole alkaloids from fungi **(B)**.

Filamentous fungi are known prolific producers of SMs. The fungal kingdom currently consists of around 120,000 identified species, but this number is estimated to represent only 3%–8% of the predicted number of existing species in our biosphere (Hawksworth and Lücking, 2017). Thanks to next-generation and third-generation sequencing technologies, in recent years the number of publicly available genomes has grown tremendously. As of this moment (early 2022), there are several thousand fungal genomes deposited in public databases, e.g., more than 2000 only on Mycocosm, a project maintained by the Joint Genome Initiative (JGI) (Grigoriev et al., 2014). The simultaneous development of automated genome mining tools such as antiSMASH (Blin et al., 2021) and other bioinformatics tools (Kautsar et al., 2019; Navarro-Muñoz et al., 2020) allowed researchers to identify a vast and unknown biosynthetic potential within the fungal kingdom in the form of SM encoding biosynthetic gene clusters (BGCs) (Figure 1A) (Mosunova et al., 2020). Bioinformatic analysis of 1,037 fungal genomes from the Ascomycota, Basidiomycota, and non-Dikarya revealed that the number of BGCs per genome significantly varies across fungal genomes (Robey et al., 2021). In the Ascomycota phylum Pezizomycotina genomes harbor on average 40 SM BGCs (25% of the genomes within this class possess >60 BGCs), however, this number is significantly lower in non-Dikarya (∼15 BGCs), in Basidiomycota (<10 BGCs) or non-Pezizomycotina Ascomycota genomes (∼5 BGCs) (Robey et al., 2021). Because of the richness and diversity of SM BGCs contained within their genomes, Pezizomycotina fungi are by far the most studied taxon in the field of SM discovery. Unfortunately, most of the BGCs encoded in their genomes are transcriptionally silent under laboratory cultivation conditions (Keller, 2019).

Fungal SM BGCs can be activated *via* manipulation of cultivation conditions or by genetic modifications. Using different cultivation conditions or co-cultivation with other organisms (Bode et al., 2002; Oh et al., 2007) has led to successful examples of BGC activation, as we further discuss in Section. Replacement of the promoter driving the expression of local or global transcriptional regulators is a commonly used genome editing strategy for transcriptional activation, e.g., overexpression of transcriptional activators or knock-outs of transcriptional repressors, as well as manipulation of epigenetic modulators, which function as global chromatin regulators (Brakhage and Schroeckh, 2011). Traditional metabolic engineering methods combined with the implementation of the “clustered regularly interspaced short palindromic repeats” (CRISPR) technologies (Doudna and Charpentier, 2014) further accelerated strain construction and enabled more complex and sophisticated genetic engineering of filamentous fungi (Song et al., 2019). BGCs can be transcriptionally upregulated by conventional genome editing approaches, but thanks to the latest developments in synthetic biology, new attractive genetic tools have become available: synthetic transcription factors (STFs), artificial transcription units, fungal shuttle vectors, and various enhanced platform strains for heterologous expression. In the following sections we present recently developed tools and discuss how they compare to each other and to conventional metabolic engineering approaches.

### Fungal Secondary Metabolite Biosynthetic Gene Clusters

SMs are low molecular weight, structurally heterogeneous compounds–synthesized by bacteria, fungi, and plants–which are not directly involved in the normal growth, development, or reproduction of the organism. SMs are synthesized from metabolic intermediates from primary metabolism. The produced SMs commonly provide a biological advantage to their producers, to thrive and survive in their environment, for instance in supporting the competition against other organisms (toxins and antimicrobials) and in the protection against harsh environments (pigments and iron-chelating siderophores), but SMs are also used for chemical signaling (Spiteller, 2015). Although many SMs have no known function, these compounds probably fulfill a role in complex communication networks in ecosystems, but so far it is just a human interpretation with limited experimental evidence.

The core skeleton of fungal SMs is produced by dedicated biosynthetic enzymes that belong to a few distinct families: nonribosomal peptide synthetases (NRPS), polyketide synthases (PKS), terpene synthases (TPS), dimethylallyltryptophan synthases (DMATS), or combinations thereof (Keller, 2019). NRPS and PKS are complex multi-modular megaenzymes that utilize a variety of amino acids and acyl-CoA monomers as substrates, respectively. TPS and DMATS are generally smaller and use a more limited set of substrates: the former use intermediates of the mevalonate pathway (IPP and DMAPP) as starter units for terpene synthesis (Keller, 2019); the latter utilize DMAPP to prenylate the amino acid tryptophan or other aromatic substrates (Kremer and Li, 2008). In all cases, the core scaffold is generally further modified by tailoring enzymes (oxidases, reductases, methyltransferases, cytochrome P450 monooxygenases, and others) whose genes are often found in the same BGC, ensuring a broad chemical diversity of the products (Figure 1B). Furthermore, these clusters frequently contain genes encoding transporters and regulatory proteins (Figure 1A). The size of a BGCs can span from a few kb to ∼100 kb incorporating as little as only two genes (valactamide BGC) (Clevenger et al., 2017) or up to ∼27 genes (aflatoxin BGC) (Caceres et al., 2020). Given that they are the most abundant in filamentous fungi—particularly in more commonly studied members of Pezyzomycotina—we will mainly discuss NRPS- and PKS-encoding BGCs in the remainder of this section.

NRPS enzymes synthesize a broad class of small peptides, typically 2–50 monomers from a wide variety of amino acids and their derivatives, as well as fatty acids and alpha hydroxy acids (Süssmuth and Mainz, 2017). These enzymes have a modular structure, where each module is responsible for the activation and coupling of a monomer to a growing peptide chain. A minimal NRPS module consists of an adenylation (A), condensation (C), and thiolation (T) domain (also called peptide-carrier protein). The A domain recognizes the monomer substrate and activates it as an (amino) acyl-AMP conjugate, which is subsequently transferred to the T domain *via* a transesterification reaction. The activated substrates/intermediates are then transported to the C domain, which is responsible for the formation of the peptide bond. Eventually, the final product is released by a terminal thioesterase domain (Fischbach and Walsh, 2006; Süssmuth and Mainz, 2017).

Polyketides represent the most abundant group of SMs. PKS enzymes utilize activated short-chain organic acids derived from primary metabolism, such as acetyl-, malonyl- or methylmalonyl-coenzyme A, for the biosynthesis of polyketides. The basic set of domains consists of an acyl carrier protein (ACP), a β-ketoacyl synthase (KS), and an acyltransferase domain (AT). The AT domain selects and loads both starter and extender monomers, while the KS domain catalyzes C-C bond formation between two adjacent substrates/intermediates. The ACP domain is responsible for storing and shuttling monomer substrates and synthesized intermediates during the elongation process. PKS enzymes are extremely diverse, many contain optional domains that introduce further chemical modifications, generating an incredible variety of products (Fischbach and Walsh, 2006; Robbins et al., 2016).

Molecules can be also constructed from hybrid NRPS-PKS assembly lines leading to mixed NRP-PK products, such as the bacterial bleomycin, rapamycin, epothilones, or the fungal fusarin C, pseurotin A, tenellin, and cytochalasin E. (Fischbach and Walsh, 2006; Boettger and Hertweck, 2013). NRP-PK hybrids can be synthesized by proteins containing domains and modules from both PKSs and NRPSs organized in the same polypeptide chain (tethered), but these enzymes can also be formed from individually expressed proteins in the BGC (non-tethered) (Miyanaga et al., 2018). In these hybrid systems, the different subunits need to communicate efficiently to coordinate the transport of substrates and intermediates across the hybrid system, and have to perform either C-C or C-N bond elongations at the corresponding PKS/NRPS interfaces (Fischbach and Walsh, 2006).

BGCs encoding NRPS and PKS clusters can readily be predicted and identified from genomic data by advanced bioinformatics algorithms, for example, using the conserved domains of the core enzymes. Such tools are the “Secondary Metabolite Unique Regions Finder” (SMURF) (Khaldi et al., 2010) or the “antibiotics and secondary metabolite analysis shell” (antiSMASH) (Blin et al., 2021). AntiSMASH is continuously updated since its release in 2011, incorporating several newly developed algorithms, e.g., searching for shared transcription factor (TF) binding sites in the promoter sequences [“Cluster Assignment by Islands of Sites” (CASSIS) (Wolf et al., 2016)]. Cross-referencing with public databases further aids the identification of uncharacterized BGCs. One example is the MIBiG (Minimum Information about a Biosynthetic Gene cluster) repository, which contains curated information in a unified format listing the BGC annotations and their molecular products (Kautsar et al., 2019). As these algorithms accept annotated DNA sequences as input, cluster predictions can be further advanced taking into account transcriptome data of the predicted BGC, assuming that the cluster can be brought to a transcriptionally activated state. Algorithms such as MIDDAS-M (motif-independent *de novo* detection algorithm for SM BGCs) aim to combine genomic data and transcriptomic data to predict coordinately regulated genes including fungal BGCs (Umemura et al., 2014). As homologues of clusters with known compounds can be easily identified, the use of databanks and algorithms can reduce the re-discovery rates or yield predictive information regarding the targeted BGCs. The discovery of a huge number of transcriptionally silent BGCs through bioinformatics fueled the interest in genome mining, and the interrogation of these unknown clusters by experimental identification (Nielsen et al., 2017; Caesar et al., 2020).

### Regulation and Transcriptional Activation of Biosynthetic Gene Clusters

SM production is often regulated by a stimulus, and without it, the product of the BGC is not synthesized. When no known metabolite is connected to a BGC, the cluster is called “cryptic” or “orphan.” In most cases, BGCs react to various environmental stimuli, but often the connection between regulators and the stimuli is unknown. Under laboratory conditions, native environmental signals may not be present, rendering BGCs transcriptionally silent. Since cryptic BGCs appear to be silent under laboratory conditions (Keller, 2019), alternative strategies need to be employed to awaken these clusters and explore their biosynthetic potential.

Conventional methods for transcriptional activation of genes or even entire BGCs have been rapidly implemented in fungal biotechnology (Brakhage and Schroeckh, 2011; Lim et al., 2012). One of the strategies concerns the OSMAC (one strain many compounds) approach, which assumes that one strain is capable to produce numerous compounds, but different environmental or cultivation conditions regulate what subset of BGCs are activated (Bode et al., 2002). Indeed, modifications for cultivation parameters such as temperature, salinity, aeration and others, showed that *Aspergillus ochraceus* is capable of producing 15 compounds in addition to the previously known aspinonene (Bode et al., 2002). Co-culturing can also result in transcriptional activation of BGCs due to inter-species crosstalk (Oh et al., 2007; Bertrand et al., 2014; Netzker et al., 2018): co-cultivating *A. nidulans* with the soil-dwelling bacterium *Streptomyces rapamycinicus* resulted in the production of orsellinic acid (Schroeckh et al., 2009). Co-cultivation of *A. fumigatus* with the same bacterium resulted in the activation of the fumicycline BGC, which involved epigenetic regulation changes induced by the bacterium (König et al., 2013).

### Global Regulators

Around half of the fungal BGCs do not harbor in-cluster regulators, and are only regulated by global transcription regulatory mechanisms (Keller, 2019). Global transcriptional regulators respond to environmental stimuli by coordinated up or downregulating of required gene sets, and the corresponding TFs to these signals have been identified in several cases: CreA responds to carbon levels; the velvet complex to light; AreA to nitrogen concentration; PacC to pH levels; and the CCAAT-binding complex to iron concentration (Macheleidt et al., 2016). These regulators act genome-wide on numerous genes, controlling morphological development, primary metabolism as well as SM production. Both overexpression (e.g., LaeA transcriptional activator of secondary metabolism) (Bok and Keller, 2004) and deletion of master regulators (e.g., McrA repressor protein) (Oakley et al., 2017) resulted in transcriptional activation of BGCs.

### Chromatin-Mediated Regulation

Alterations in the structure of chromatin can result in global transcriptional regulatory effects (Collemare and Seidl, 2019). Histones are critical proteins responsible for the tight packing of DNA in the nucleus, creating the chromatin. Histones can undergo numerous modifications (acetylation, methylation, phosphorylation, uniquitination, sumoylation, and neddylation), and this can create less- or more accessible DNA segments (Pfannenstiel and Keller, 2019). Histone deacetylation results in a more closed chromatin structure causing transcriptional repression of affected genes. In contrast, histone acetylation can result in more accessible chromatin for regulator proteins and the transcription machinery, causing transcriptional activation. The structure of chromatin can be manipulated by using chemical agents or genetic modifications to achieve transcriptional regulation of genes of interest. For instance, chemical histone deacetylase (HDAC) inhibitors (e.g., suberoylanilide hydroxamic acid, trichostatin, and sodium butyrate) can be supplemented to the cultivation media to prevent histone deacetylation (Pfannenstiel and Keller, 2019). Such epigenetic perturbation may lead to a 100-fold up and downregulation of genes that are spread over the genome (Albright et al., 2015). Epigenome editing for transcriptional activation is also possible by genetic engineering of the regulation of the expression of histone acetyltransferase (HAT) or HDAC genes. In *A. nidulans*, downregulation of the gene *rpdA* that encodes a HDAC enzyme yielded similar results as observed with chemical HDAC inhibitors (Albright et al., 2015). Deletion of the *hdaA* (histone deacetylase A) in *A. nidulans* resulted in increased penicillin and sterigmatocystin production (Keats et al., 2007). In *P. rubens* [previously identified as *P. chrysogenum* (Houbraken et al., 2011)], deletion of a *hdaA* homolog positively affected the production of sorbicillins and roquefortine/meleagrin (Guzman-Chavez et al., 2018), and significantly downregulated the BGCs responsible for chrysogine and dihydroxynaptelene-melanin production (Ding et al., 2020).

### Cluster Specific Regulators

Around half of the predicted BGCs harbor genes encoding TFs, which are often transcriptional activators of the complete cluster (Lyu et al., 2020). These regulators bind to the corresponding recognition sequence in the promoters of the genes in the BGC. As promoter replacement is a good strategy to override the native regulation of a transcriptionally silent gene, replacing the promoter of the in-cluster TF can result in cluster-specific activation. Although overexpression of cluster specific TFs has led to the production of aspyridones, asperfuranone, and emodin derivatives in several Aspergilli (Bergmann et al., 2007; Chiang et al., 2009; Lyu et al., 2020), a systematic promoter replacement approach in *A. nidulans* showed that only 3 out of 17 overexpressed cluster specific TFs effectively led to the production of an obtainable amount of SMs (Ahuja et al., 2012). Although inducers and protein-protein interactions affect the activity of the TF, it is currently unknown what other mechanism(s) are required for complete BGC activation alongside the overexpression of an in-cluster TF. Cross-talk between different cluster specific TFs have also been described in *A. nidulans*, as the overexpression of the ScpR TF (from the fellutamide BGC) caused upregulation of the in-cluster *inpA* and *inpB* NRPS genes, as well as the asperfuranone BGC, located on a different chromosome (Sebastian et al., 2010). Since the AflR (aflatoxin transcriptional activator) recognition sequence can be found in most of the promoters in the sterigmatocystin and aflatoxin cluster, this TF was shown to be able to regulate positively both BGCs, as well as some genes outside of these BGCs (Brodhagen and Keller, 2006; Price et al., 2006).

In-cluster SM BGC transcriptional repressors with DNA-binding capacity have so far not been discovered in filamentous fungi. Rather, repressor proteins interacting with transcriptional activators is a more common mechanism. For example, the primary metabolism BGC responsible for the quinic acid degradation in *N. crassa* is controlled by a transcriptional activator/repressor regulator pair (qa-1F/qa-1S) (Giles et al., 1991). Similarly, in *A. niger* the repression of galacturonic acid utilization pathway is modulated by a regulator pair (Gaar/GaaX) (Niu et al., 2017). It is believed, that these in-cluster repressors are responsible for keeping the positive transcriptional regulator inactive in the absence of an inducer (Giles et al., 1991; Niu et al., 2017). The sorbicillin SM BGC in *P. rubens* harbors an activator/repressor pair as well, and the metabolites of the cluster are acting as autoinducers for the pathway (Guzmán-Chávez et al., 2017). Overexpression of the transcriptional repressor (*sorR2*) results in transcriptional suppression, while deletion of *sorR2* results in early-stage transcriptional activation of the sorbicillin BGC, but with hardly any sorbicillin production (Guzmán-Chávez et al., 2017). Although promoter replacement, gene deletion or complete BGC refactoring in the native host leads to direct transcriptional activation of the gene of interest, but these methods require editing the genome of the fungus.

### Fungal Genome Editing

Precise and flexible genome editing is key for efficient engineering of the fungal host. Targeted gene manipulation in wild type filamentous fungal species is challenging due to the relatively low rates of homologous recombination (HR) and high rate of random integration of the transformed DNA. Targeting efficiency to the desired location is relatively low in *Aspergillus* and *Penicillium* species (0.1%–5.0%) (da Silva Ferreira et al., 2006; Snoek et al., 2009), and it differs by the organism and targeted locus. The fungal homologs of the human *ku70/ku80* genes encode a protein complex functioning in the non-homologous end-joining (NHEJ) DNA repair pathway, which favors random integration of the transformed donor DNA. Deletion of either of these genes is highly advantageous for fungal strains that are employed for precise genome editing and HR-mediated DNA delivery. Inactivation of either of the fungal homolog of the human *ku70/ku80* genes drastically decreases or eliminates the functionality of the NHEJ DNA repair pathway and highly increases the efficiency of targeted DNA delivery through HR (Pöggeler and Kück, 2006; Takahasi et al., 2006; Snoek et al., 2009; Carvalho et al., 2010). With the advent of CRISPR-based tools the genome editing efficiency was further increased, in some filamentous fungal strains reaching more than 90% (Pohl et al., 2016; Schuster et al., 2016; Nødvig et al., 2018; Song et al., 2018).

A commonly used alternative to engineer fungal hosts is the *Agrobacterium*-mediated transformation, particularly employed when little or no genetic tools are available for that host. *A. tumefacians* is a gram-negative plant pathogen that has been shown to be capable of transferring its T-DNA (transfer-DNA, used by the bacteria to infect plants) into the genome of several filamentous fungi. This made it possible to achieve successful DNA delivery in fungal hosts that cannot be transformed with traditional methods (de Groot et al., 1998; Idnurm et al., 2017). The system is commonly used for random integration of one single copy of a gene of interest, but it can also be used for targeted genome editing in NHEJ-deficient hosts *via* homologous recombination using long homologous flanking sequences (>1000 bp) (Idnurm et al., 2017).

Before the global application of CRISPR-based genetic tools, zinc finger nucleases (ZFNs) and transcription activator-like effector (TALE) nucleases were established for locus-specific genome engineering applications. Engineering the DNA binding domain (DBD) of these nucleases allows targeting specific genomic loci. ZFNs are artificial restriction enzymes and are typically generated by fusing DBSs with the FokI nuclease domain. DNA targeting is provided by fusing together three to six DNA-binding zinc-finger proteins, each of which is capable to recognize a specific 3 bp DNA sequence. Although ZFNs are relatively small proteins, which are easy to deliver to the host, their targeting efficiency is rather weak and the relatively high levels of off-target effects may lead to cytotoxicity (Ramalingam et al., 2011). The next generation of targeted DNA editing was the discovery of the transcription activator-like effector nuclease (TALEN) elements, which are acting as TFs in the species of *Xanthomonas*. The DBD builds up from 33 to 34 amino acid long tandem repeats, which determines the targeted DNA sequence. These repeats can be altered to recognize one specific nucleotide and by combining these repeats in sequential order, the protein can be targeted to any DNA sequence (preceded by a thymine or cytosine base). Direct fusion of the TALE DBD and with restriction endonuclease (FokI) domain created guidable TALENs, meanwhile fusions to transcription activation domains (ADs) to created STFs for targeted transcriptional regulation (TALE-TFs) (Gao et al., 2014).

### CRISPR-Mediated Genome Editing

The CRISPR systems and their CRISPR-associated (Cas) proteins have recently been repurposed for transcriptional gene regulation in eukaryotes (Gilbert et al., 2013; Perez-Pinera et al., 2013), where they can be utilized as components of STFs. Native Cas proteins provide a self-defense mechanism against bacteriophage virus infection in prokaryotes. When these organisms encounter the virus for the first time, they embed small viral sequences in their genome, which are later transcribed into small RNAs molecules. These RNAs form complexes with the Cas proteins, which are now able to recognize and cleave the complementary nucleic acid sequence in the viral genome at any next infection event, effectively eliminating the intruder (Rodolphe et al., 2007). Cas proteins commonly cleave double or single stranded DNA, but RNA-cleaving Cas proteins have also been identified (Knott and Doudna, 2018). Repurposing and utilizing these systems for targeted genome editing has revolutionized precise genome engineering in various organisms. In these two component CRISPR systems, the Cas protein is guided by a CRISPR RNA (crRNA) to a target specific locus for nucleic acid cleavage. For the commonly used Cas9 systems, specificity is delivered on a single guide RNA (sgRNA) complex that encodes both the short trans-activating CRISPR RNA (tracrRNA) and crRNA transcripts. The tracrRNA forms the stem loops that anchor the endonuclease protein while the crRNA is the actual targeting sequence. In contrast, the other commonly applied Cas12a (Cpf1) nuclease is capable to processes its own crRNAs from pre-crRNA, and does not need a tracrRNA (Fonfara et al., 2016).

With the Cas9 system, the genomic locus is targeted by a sequence-specific 17–20 nucleotide crRNA which is complementary to its genomic target (protospacer), that must be followed by a protospacer adjacent motif (PAM). This PAM sequence is recognized at the DNA level by the protein and is unique for different Cas proteins. These unique PAM sequences limit the number of sequences that one can target since they show minimal flexibility for different nucleotides: For example, the PAM sequence recognized by the commonly used *Streptococcus pyogenes* Cas9 (SpCas9) is 5′-NGG-3′ (to a lesser extent non-canonical NAG and NGA are also recognized, where N is any nucleotide) located downstream the protospacer, meanwhile the Cas12a nuclease recognizes 5′-TTTV-3′ (where V can be G, C or A) sequences located upstream of a typically 20–24 nucleotide long protospacer (Pickar-Oliver and Gersbach, 2019). Careful design of the crRNA is therefore essential to avoid off-target CRISPR effects, as the nuclease complex is capable to bind to highly similar sequences (Fu et al., 2013), which represents another limitation of this system.

In recent years, highly efficient CRISPR-based genome editing tools have been developed and established for several organisms, such as bacteria, yeast, and human cells (Wang et al., 2016). CRISPR/Cas9-based genome editing in filamentous fungi has been established for several organisms including *A. fumigatus*, *A. oryzae*, *Neurospora crassa*, *Pyricularia oryzae*, *Trichoderma reesei*, *Ustilago maydis*, and *P. rubens* (Song et al., 2019). CRISPR elements can be delivered as ribonucleoproteins preassembled *in vitro*, or as genetic elements that are expressed by the host. The AMA1 (autonomous maintenance in *Aspergillus*) sequence from *A. nidulans* supports autonomous vector replication in several filamentous fungal species (Fierro et al., 1996; Aleksenko and Clutterbuck, 1997), and thus vectors encoding this sequence have been extensively used for gene delivery and expression purposes, as well as delivering CRISPR components (Fuller et al., 2015; Nødvig et al., 2015; Pohl et al., 2016; Salazar-Cerezo et al., 2020; Iacovelli et al., 2021b). Single vector-based CRISPR/Cas9 genome editing systems have been previously developed for several filamentous fungal species (Song et al., 2019). Recently, a similar system was developed based on the nuclease Cas12a (Cpf1) for Aspergilli (Vanegas et al., 2019).

## Fungal Synthetic Biology Tools

Synthetic biology has revolutionized metabolic engineering with tools–created by repurposing or redesigning biological systems found in nature–enabling the exploitation of industrial microorganisms at whole new levels. Since synthetic biology strives to engineer highly predictable and controllable genetic systems, genetic circuits are often constructed in a standardized and preferably modular fashion. The modularity of various DNA parts encoding genetic elements allows rapid assembly of novel, more predictable genetic circuits, like logic gates, and genetic switches. Inducible or synthetic transcriptional regulators (activators and repressors) can be used to enable fine-tuning of gene expression or controlling entire pathways. Synthetic biology-based tools have been established in several model bacterial and eukaryotic systems, and recently also in filamentous fungi where they are still relatively underdeveloped compared to more common hosts.

### Modular Assemblies

The Design-Build-Test-Learn (DBTL) cycle of synthetic biology represents a systematic and efficient workflow for the optimization of biological systems for speciffic functionalities (e.g., strain improvement). Complex genetic systems can be constructed in a modular manner with desired features–synthetic regulatory tools and rewired expression of biosynthetic pathways–enabling an affordable genetic engineering of biological systems. Synthetic transcription units can be rapidly assembled by cloning methods supporting the assembly of multiple DNA fragments [e.g., Gibson Assembly (Gibson et al., 2009) or USER Cloning (Geu-Flores et al., 2007)], or high-throughput, modular cloning methods, such as Golden Gate cloning-based (Engler et al., 2009) Modular Cloning (Weber et al., 2011), and GoldenBraid (Sarrion-Perdigones et al., 2011) assemblies. With these methods, genetic parts (promoters, coding sequences, and terminators) can be arranged into transcription units, where the building blocks are interchangeable within the same synthetic biology language, but their order and orientation are commonly predetermined. Collections of such DNA building blocks (toolkits) have been established for bacteria (Moore et al., 2016), yeasts (Lee et al., 2015; Obst et al., 2017), plants (Engler et al., 2014), mammalian host cell lines (Martella et al., 2017) and also for filamentous fungi (Sarkari et al., 2017; Hernanz-Koers et al., 2018; Mózsik et al., 2021b; Dahlmann et al., 2021). These toolkits can provide backbone vectors to facilitate modular assembly and fungal delivery, or pre-assembled vectors containing various genetic elements, suitable for generic applications or specific needs. The deposition of such toolkits containing ready-to-use, established DNA parts or modules, highly accelerates the biological DBTL cycle for synthetic biology applications. A major repository for genetic parts is Addgene, a free online database that facilitates the exchange of genetic material between laboratories around the world.

### Artificial Promoters and Synthetic Transcription Factors

Eukaryotic promoters are complex DNA structures responsible for recruiting transcriptional regulatory elements (transcriptional regulators). The simplest functioning unit of the promoter—often called minimal or core promoter (CP)—is incorporating the transcription start site (TSS) and is required to initiate transcription of the gene of interest. CPs contain specific DNA elements that the RNA polymerase II requires to initiate transcription (Michael, 1998). In eukaryotes, CP sequences are highly diverse: many motifs can be present such as the TATA, CCAAT boxes, the B recognition element, and the initiator element (Juven-Gershon and Kadonaga, 2010). Several regulatory TF-binding sequences are located upstream of the CP sequence, where they recruit transcriptional activator or repressor proteins, hence, modulating the transcription of the gene. The length of CPs is not well defined in filamentous fungi, but these sequences are located roughly 140–200 bp upstream of the starting codon (Rantasalo et al., 2018; Mózsik et al., 2019). The precise identification of upstream regulatory DNA sequences and CPs is essential for the engineering of functional synthetic promoters.

Synthetic gene expression systems can be used for the production of metabolites or proteins of interest, and have been established in numerous filamentous fungal species (Vogt et al., 2005; Rantasalo et al., 2018; Mózsik et al., 2019). Such orthogonal systems do not rely on the regulatory system of the host, but instead depend on hybrid or synthetic TFs, composed of different DNA-bindings (DBDs) and transcriptional effector domains, and on synthetic promoters. DBDs target and bind to unique upstream activating sequences (UASs) in the promoter region, effectively regulating gene expression. If the TFs are inducible and/or repressible upon the addition of small molecules, these systems can be used as genetic switches as well. STFs have been repurposed from different prokaryotic, eukaryotic, or viral transcriptional regulators and have been shown to be functional in several hosts including yeast and filamentous fungi. Using such synthetic transcriptional regulators, activation or repression of genes can be achieved in a controlled manner. Transcription can be fine-tuned for each gene individually by changing different elements of the system. Synthetic promoters created by fusing specific UAS and CPs, or by integrating UAS elements into native promoters, can be used to rewire the native transcriptional regulation system of the genes of interest. Synthetic promoters bring the promise of a pre-defined, fine-tuned, and metabolism-independent expression for multiple individual genes. Synthetic promoters in combination with an inducer-dependent STF can allow further tuning of gene expression in a gene dosage and/or inducer concentration dependent manner (Meyer et al., 2011). Such refactoring would allow the overexpression of entire BGCs bypassing the need for established strong promoters for each gene of the cluster, since the number of such promoters is limited for filamentous fungal hosts. Functional STFs have been successfully introduced in Aspergilli (Vogt et al., 2005; Wanka et al., 2016; Grau et al., 2018; Rantasalo et al., 2018), *P. rubens* (Mózsik et al., 2019), *T. reesei* (Derntl et al., 2019), and *Ustilago maydis* (Zarnack et al., 2006).

Many STFs have been constructed to regulate genes in primary metabolism (Gao et al., 2017; Zhang et al., 2018; Han et al., 2020; Kun et al., 2021; Yamashita et al., 2021). Fusing the DNA-binding domain of the CreA/Cre1 (carbon catabolite repression) transcription factor to the complete Xyr1 transcription factor (Xylanase Regulator 1) resulted in enhanced cellulase production in a CreA/Cre1 deficient *T. reesei* strain grown on glucose (Zhang et al., 2017). Fusing the DBD of the Xyr1 with the regulator domain of Ypr2 (transcriptional activator of the sorbicillinoid SM BGC) resulted in high expression of xylanases and cellulases in *T. reesei* nearly independently from the carbon source used (Derntl et al., 2019). Replacing the regulatory domain of a weak in-cluster transcriptional TF with a highly active activator domain (AD) can lead to activation of target SM BGCs, without the integration of additional synthetic promoter elements. When the DNA-binding domain of the transcriptional activator (AlnR) from the asperlin BGC was fused to the regulatory domain of the transcriptionally highly active asperfuranone TF (AfoA), it led to the production of asperlins in *A. nidulans* (Grau et al., 2018). In these works, the DBD in the STF retained its capability to bind to its native operator sequences in the promoters, while the newly fused activator domain provided transcriptional activation of the genes.

Although STFs (also called altered, artificial, or hybrid TFs) have been studied for more than 30 years (often using the Gal4 TF as a model from *Saccharomyces cerevisiae*) (Ma and Ptashne, 1987; Corton, 1989; Hach et al., 2000), there is limited information about how these domain fusions should be engineered to avoid creating nonfunctional STFs. Cluster-specific TFs commonly consist of a Zn(II)_2_Cys_6_ (C6 zinc) DBD and a transcriptional regulator domain. DBDs often contain at least one structural motif that recognizes and bind to double- or single-stranded DNA sequences. Generally, DBDs can be further divided into sub regions: the zinc finger, the linker region, and a coiled-coil element. Numerous Gal4-family TFs contain a coiled coil between the linker and the regulator domain, which is possibly responsible for mediating protein-protein interactions or homodimer formation before binding to DNA (Hach et al., 2000). While structural changes in the zinc-finger motif, the linker region or the coiled-coil regions negatively affect the functionality of the TF, the regions between these coiled-coil sequences and the regulator domains are often non-essential for retaining activity (Ma and Ptashne, 1987; Corton, 1989; Marmorstein and Harrison, 1994).

The tetracycline-inducible (TET) expression system has been originally developed for mammalian cells (Gossen and Bujard, 1992), and later adopted for other eukaryotic systems, as well as for Aspergilli and *U. maydis* (Vogt et al., 2005; Zarnack et al., 2006; Meyer et al., 2011). Within the endogenous tetracycline-resistance system in Gram-negative bacteria, the TetR transcriptional repressor represses the expression of the tetracycline transporter gene (TetA) by binding to the TetO (or “tetracycline response element” TRE) operator sequences in the promoter. In the presence of the antibiotic tetracycline, TetR will bind the compound and be released from TetO, enabling expression of the transporter gene which eventually provides self-resistance (Orth et al., 2000). This repressor was engineered into an activator in the Tetracycline-Off (Tet-Off) system, where a tetracycline-controlled STF, the tTA (TetR-VP16 fusion) provides inducible repression. Transcriptional repression can be achieved by feeding tetracycline (or its synthetic derivative doxycycline) to the medium, which binds to the synthetic activator (tTA), thus preventing binding to the TetO sequences and the expression of the gene of interest. Several copies of the TetO sequences are inserted upstream of a weak or transcriptionally silent minimal (core) promoter for transcriptional regulation of the gene of interest. As tetracycline and doxycycline have relatively short half-lives, these chemicals need to be added to the medium repeatedly to maintain transcriptional repression, and in their absence transcriptional activation occurs, as the tTA binds to the TetO sequences.

Using the further engineered Tetracycline-On (Tet-On) system, gene activation can be achieved in a concentration-dependent manner by feeding the inducer and using the reverse tetracycline-controlled transcriptional activator (rtTA, mutated TetR-VP16 fusion) as the STF. In the presence of tetracycline (or doxycycline), the affinity of this rtTA STF towards the TetO sequences increases, therefore enhancing the transcription of the gene of interest downstream. Unfortunately, the rtTA retains some binding affinity to its TetO sequences in the absence of the inducer, leading to leaky transcription. Thus, an advanced version of rtTA (rtTA2^S^-M2, TetR-3xVP16) was designed, showing increased specificity, stability, and inducibility using doxycycline without leaky expression (Urlinger et al., 2000). This Tet-On system was applied with *A. fumigatus* for inducible expression of the gene of interest using seven copies of the TetO sequence upstream a short 175 bp CP sequence of the commonly used *gpdA* promoter (Vogt et al., 2005). The system was established in *A. niger* using fluorescent reporters, and was applied for the production of fructose-6-phosphate amidotransferase (Wanka et al., 2016) and biologically active fungal cyclodepsipeptides (Enniatin B, Beauvericin, Bassianolide) on a grams-per-liter scale (Boecker et al., 2018).

The bacterial Bm3R1-based STF (Bm3R1-DBD-VP16) was shown to be functional for transcriptional activation in yeasts as well as in *A. niger* and *T. reesei* (Rantasalo et al., 2018). This STF was delivered to different fungal hosts harboring several copies of the BS-UAS, enhancing the transcription capacity of various, native and non-native CPs to control gene expression in fungi. These results showed that, although CPs function differently among hosts, universally functional CPs which operate both in filamentous fungi and yeast hosts can be designed. Some of these synthetic promoters even performed better than commonly used native “strong” promoters. As the native TFs have no known inducers, controlling or inducing the transcription in this system is not established.

The transcriptional activator and repressor of the quinic acid metabolism from *Neurospora crassa* (Giles et al., 1985) have been implemented as a binary expression system for *Drosophila melanogaster* and mammalian cell lines, known as the “Q-system” (Potter et al., 2010)*.* This system has controllable features as in the native host the repression of qa-1F by the qa-1S transcriptional repressor can be relieved by feeding with quinic acid, resulting in inducible activation. In the earliest example of an engineered Q-system, a STF was constructed by fusing the DBD of the qa-1F transcriptional activator to the GAL4 AD. This DBD binds to its corresponding recognition sequences upstream of the targeted promoter (called QARE QA response element or QUAS Q-System UAS) (Giles et al., 1985; Potter et al., 2010). The Q-system was later also adapted and established for mammalian cells, *Caenorhabditis elegans*, zebrafish, and malaria mosquitos (Riabinina et al., 2016). Based on the Q-system transcriptional activator (qa-1F), a STF using the VP16 AD (qa-1F-DBD-VP16-GFP) has been constructed in *P. rubens*, where the strength of the Q-system STF device showed scalability by using different CPs, by increasing the expression levels of the STF or the number of UAS elements (1, 5 or 11) upstream of the CP (Mózsik et al., 2019). The system was capable to produce expression levels ranging from hardly detectable to a level similar to that of highly expressed native genes. These synthetic expression devices were validated using fluorescent reporters while the application potential was confirmed by synthetically controlling the expression of the penicillin BGC. The development of such a system further increased the number of genetic regulation tools available for filamentous fungi.

### CRISPR-Based Transcriptional Regulation

Mutations in the nickase domain(s) of the CRISPR protein eliminate its nuclease activity while retaining the capability of the protein to bind to the DNA. Such “nuclease-dead” CRISPR proteins (dCas) are engineered from Cas9 by introducing point-mutations in the RuvC and HNH nuclease domains (in dCas9m2 from *S. pyogenes* these are the D10A and H840A, respectively). Similarly, point mutations are introduced in the RuvC-like domain of Cas12a to generate its corresponding dCas variant (E993A in dCas12a from *Acidaminococcus* sp.) (Yamano et al., 2017). These proteins can be fused to ADs and used as STFs (CRISPRa, activation), thereby recruiting a transcriptional regulator to the promoter of the gene of interest. Since dCas proteins can still bind tightly to their target sequences, they can be guided to regions upstream of a gene of interest where they form a “road-block” for the transcriptional machinery, resulting in transcriptional repression (CRISPRi, interference). Taking advantage of the guidable DNA-binding capability, these dCas proteins can be applied for various other applications depending on the delivered modulator, e.g., targeted DNA modifications (e.g., methylation), transcriptional regulation, fluorescent imaging can be achieved (Pickar-Oliver and Gersbach, 2019). Inactivated Cas proteins can be used to deliver transcriptional regulator domains to the promoter of the gene of interest by direct fusion of regulatory domains, or repetitive peptide epitopes that recruit multiple copies of antibody-fused regulators (SunTag), or by using MS2 RNA stem-loops in the sequence of the tracrRNA to recruit MS2-tagged regulators (Synergistic Activation Mediator “SAM” system) (Konermann et al., 2015; Chavez et al., 2016).

The protospacer sequence is crucial in CRISPRi/CRISPRa applications for targeted repression and activation, respectively. CRISPRi has been successfully adapted to several bacterial and eukaryotic hosts for targeted gene repression (Farzadfard et al., 2013; Gilbert et al., 2013, 2014; Qi et al., 2013; Rock et al., 2017; Sato’o et al., 2018; Román et al., 2019; Lauren et al., 2022; Tan et al., 2022). Targeting in close distance to the TSS of the gene of interest with this system leads to successful downregulation, presumably by blocking transcriptional initiation or elongation. In prokaryotic CRISPRi applications, the bare dCas9 without any fused regulator domain is already capable of achieving significant repression. It is believed that the binding of dCas9 can hinder the binding of positive enhancers or the mediator complex for transcriptional elongation. In eukaryotes, the levels of repression achieved by using dCas9 alone are low, but can be enhanced by fusing repressor domains such as KRAB (Krüppel associated box) or Mxi1 (a histone deacetylation mediator) (Qi et al., 2013). The efficiency of CRISPRi-based repression differs depending on several factors including the type of fused effector, off- and on-target effects of the CRISPR protein, the distance of the protospacer from the TSS, and the chromatin state of the target genomic region (Smith et al., 2016). Presumably, the native transcription levels of the target genes and the presence of regulatory protein binding sequences in close proximity of the CRISPR complex also affect the degree of repression. In mammalian cell lines, the dCas9-KRAB fusion provides repression when targeted in the range of −50 to +300 bp relative to the TSS of a gene, with the highest efficiency of ∼100-fold repression in the −50 to +100 bp region (Gilbert et al., 2014). In *S. cerevisiae*, the dCas9-Mxi1 fusion resulted in a maximal ∼10-fold repression when targeted to the −200 to +1 region relative to the TSS, but this reduced efficiency could be explained by the mode of repression employed by Mxi1, which mediates DNA deacetylation (Smith et al., 2016). These experiments also highlight how nucleosome occupancy and chromatin accessibility can affect crRNA efficiency. The level of repression can be further increased by deploying multiple sgRNAs in combination with the Cas9 systems (Farzadfard et al., 2013) which can be achieved by using sgRNA-arrays in combination with self-cleaving sequences (Hammerhead and HDV ribozymes and tRNA) (Gao and Zhao, 2014; Zhang Y. et al., 2019) or exogenous nucleases and their cleavage factor recognition sequences (Csy4 nuclease) (Ferreira et al., 2018).

CRISPR-based transcriptional activation systems commonly use the VP16 AD or variants thereof where the VP16 is arranged in tandem repeats (VP64, VP160). This regulatory domain originates from herpes simplex virus, but it was shown to function in various organisms (Sadowski et al., 1988; Gossen and Bujard, 1992; Vogt et al., 2005; Rantasalo et al., 2018; Mózsik et al., 2019). VP64 was also combined with two other potent transcriptional activators to generate the VPR (VP64-p65-Rta) tripartite activator domain, which has been shown to be superior compared to other activator domains tested in human, mouse, and fly cell lines as well as in the yeast *S. cerevisiae* (Chavez et al., 2015). The Cas-VPR fusion system has been successfully adopted for filamentous fungi, and established for *A. nidulans* (Roux et al., 2020), and *P. rubens* (Mózsik et al., 2021a). In *A. nidulans*, the dCas9-VPR and dCas12a-VPR activators were expressed from an episomal vector and were guided to the transcriptionally silent *elcA* promoter of the PKS gene of the elsinochrome BGC from *Parastagonospora nodorum*, which was fused to an mCherry fluorescent reporter gene. After transcriptional activation of *elcA* was validated using fluorescence microscopy, the system was used to overexpress individual genes of the native microperfuranone BGC in *A. nidulans*, which resulted in enhanced production of microperfuranone and the identification of dehydromicroperfuranone (Roux et al., 2020). In *P. rubens,* a vector-based dCas9-VPR system was used to activate the transcriptionally silent, native *P. rubens* macrophorin BGC by activating the promoter of the transcriptional activator of the cluster (Mózsik et al., 2021a). This CRISPR activator system was validated using a transcriptionally silent CP (Mózsik et al., 2019) driving a DsRed fluorescent reporter (Mózsik et al., 2021a). Cas12a is natively able to process its own crRNAs from an array of pre-crRNAs, while Cas9 requires additional engineering for the delivery of multiple crRNAs (e.g., individual sgRNA transcription units, self-cleaving ribozyme sequences, or Csy4 endoribonuclease cleaving) (Ferreira et al., 2018). Since targeting the same promoter with multiple crRNAs shows synergistic effects in various eukaryotic CRISPRa applications (McCarty et al., 2020), dCas12a systems are superior compared to dCas9 for gene regulation purposes.

In mammalian cell lines, CRISPRa seems to be the most effective in the range of 400–50 bp upstream of the TSS (Gilbert et al., 2014). Since the genes of cryptic BGCs in filamentous fungi are often transcriptionally silent, the TSSs are not known. In this case, crRNAs can be designed to target regions close to the predicted TSS or to the start codon of the gene of interest. Both in *A. nidulans* and *P. rubens*, this approach was successfully used to achieve transcriptional activation using individual sgRNAs guiding the dCas9-VPR activator to 162–190 bp (P*elcA*) or 106–170 bp (P*penDE*) and 68–73 bp (P*macR*) region upstream of the start codon, respectively (Roux et al., 2020; Mózsik et al., 2021a). Next to the general rules to identify CRISPR protospacer candidates (selecting predicted high on-target and low off-target binding efficiency, and avoiding strong secondary RNA structures), regulatory DNA elements in the targeted promoter, as well as local chromatin organization should be considered when designing crRNA sequences.

When designing CRISPRa strategies, particular attention should be paid to prevent undesired blockages to the transcription complex. Targeting in close proximity upstream from the TSS seems favorable, but the CRISPR complex should not be too close to create physical hindrance for the transcription complex formation, and it should also bind outside of known enhancer or transcriptional regulatory elements (TATA or CCAAT box) in the promoter. Without precise knowledge of the regulatory elements in the sequence of the targeted promoter, empirical testing of crRNAs will remain necessary. In the extent of transcriptional activation achieved with CRISPRa, upregulation is dependent on the effect of native regulatory proteins as well as the native transcription level. When the CRISPRa system is correctly positioned, transcriptionally silent genes can be drastically upregulated, while enhanced activation of transcriptionally active genes is generally marginal (Strezoska et al., 2020). Problems of incorrectly positioned CRISPR guides could be potentially solved by deploying multiple spacers to the same promoter if the chosen CRISPRa/i system supports multiplexing. To conclude, with careful design CRISPRa can be applied as a targeted transcriptional activation tool for SM discovery, bypassing the need for laborious genome editing efforts.

### CRISPR-Based Chromatin Remodeling

As discussed before, the chromatin landscape plays an important role in transcriptional regulation in filamentous fungi (Bok et al., 2009; Collemare and Seidl, 2019; Pfannenstiel and Keller, 2019). Since prokaryotic Cas proteins are not suited to cope with such obstacles as nucleosomes, it is expected that nucleosome-bound DNA hinders CRISPR activity. As CRISPR-based genome editing only involves a one-time event, and as the organization of chromatin is continuously changing, it is hypothesized that these spontaneous remodeling events contribute to the efficacy of CRISPR-based editing and its widespread success and applicability in eukaryotic organisms (Isaac et al., 2016). In contrast, for achieving potent CRISPRa/i transcriptional regulation at the promoter region, a persistent binding of the regulator is likely needed, which can be negatively affected by the chromatin state.

Nucleosome maps for fungal genomes are essentially undescribed. Since the chromatin organization can change depending on the cultivation conditions, it is advised to perform mapping in the same conditions as the CRISPRa/i application is planned to be executed. Fungal nucleosome maps could potentially help to identify genomic regions obscured by nucleosomes and therefore less accessible to the transcriptional complex, as well as nucleosome-free DNA regions, which are more favorable targets for CRISPR-based applications. Nucleosome mapping has been applied for *A. nidulans* to facilitate the design of efficient protospacers for dCas9-VPR. Indeed, targeting the nucleosome-free region of a bidirectional promoter in a cryptic BGC with a single sgRNA resulted in significant transcriptional activation of genes up and downstream of the spacer sequence (Schüller et al., 2020). Further, by targeting multiple protospacers (nucleosome-free and nucleosome-bound) synergistic activation effects were observed. For targeted chromatin remodeling, a fusion of dCas9 with the core domain of the human acetyltransferase p300 (dCas9-p300Core) has been successfully employed in mammalian cells to target enhancers regions upstream of the promoter of interest. This targeted acetylation resulted in increased expression of the downstream genes (Hilton et al., 2015). Recently, the dCas9-p300Core system has been employed in *A. niger*, where three different genes were targeted individually and successfully upregulated (Li et al., 2021).

### Biosynthetic Gene Cluster Refactoring

Expression of all the relevant genes of a BGC with constitutive, inducible, or synthetic promoters—thus involving major refactoring and cloning efforts—is an effective approach to characterize cryptic BGCs. Although using filamentous fungal hosts has numerous advantages (as will be discussed later), tools and expression platforms are still underdeveloped compared to other well-established species such as *S. cerevisiae*.

The promoter replacement technology is commonly used for the overexpression of a gene of interest. Selected promoters are capable of a high transcription rate under the employed cultivation conditions. Strong promoters are often selected by using transcriptome data analysis or empirical testing. Usually, these are strong constitutive promoters responsible for the transcription of housekeeping genes or other genes that are highly expressed *in vivo,* or show inducibility (Kluge et al., 2018) [e.g., *gpdA* (ANIA_08041), *glaA* (An03g06550), *pcbC* (Pc21g21380), *40S-rps8* (An0465), *tef1* (ANIA_04218)]. Inducible promoters are found in a similar manner, but employing a well-defined chemical (alcohols, antibiotics, hormones, or carbon sources) as a potential inducer that can be added in various amounts to repress or enhance gene expression levels (Kluge et al., 2018).

Unfortunately, individual replacement of all native promoters in a large BGC with strong promoters is an elaborate and time-consuming task, further complicated by the limited availability of well characterized fungal promoters. Nonetheless, such extensive refactoring can still be attempted with a filamentous fungal host that shows a high HR rate that facilitates recombination of DNA fragments *in vivo* (Chiang et al., 2020; Pohl et al., 2020). Single promoter replacement is much more practical when it is employed to overexpress an in-cluster regulator, which in turn results in complete BGC activation with minimal engineering efforts (Bromann et al., 2012; Zabala et al., 2012). Alternatively, prior to the fungal transformation the target BGC can be pre-assembled with the chosen promoters and terminators using advanced cloning methods or hosts with high HR rate, such as *S. cerevisiae* (Kim et al., 2010). For example, the 25 kb long geodin BGC from *A. terreus* was delivered into *A. nidulans* after pre-assembly using USER fusion from 8 PCR products containing the 13 native genes, and at the same time replacing and overexpressing the transcriptional activator of the cluster (Nielsen et al., 2013). Alternatively, such large genomic segments can be captured on fungal artificial chromosomes (FACs), as discussed in the following section (Bok et al., 2015). Since fungal promoters for overexpression approaches are limited, and not every BGC contains a specific transcriptional activator to overexpress, alternative solutions are needed. Although the decreasing prices of DNA synthesis could revolutionize BGC screening by making the synthesis of entire clusters affordable, the current price levels only allow for the synthesis of smaller DNA fragments. Polycistronic expression of multiple genes has been successfully applied in filamentous fungi using only one established promoter and one terminator (Unkles et al., 2014), and this could be a potential alternative for BGC refactoring. Synthetic promoters with orthogonal STF-based regulation (discussed earlier) could be used for a scaled, tunable or coordinated expression of refactored BGCs. Such systems can be delivered *via* genomic engineering of the native host, or by using shuttle vectors and suitable heterologous hosts.

### Fungal Shuttle Vectors

Next to methods that require introducing genetic parts permanently into the genome of the host organism, vector-based, genome-editing-free alternatives are also available for gene expression in filamentous fungi. Fungal shuttle vectors allow the pre-assembly of genes of interest or complete BGCs in well-established model organisms like *Escherichia coli* or *S. cerevisiae*, thereby facilitating rapid cloning and subsequent delivery to the desired expression host. Since the isolation and identification of the AMA1 replicator sequence from *A. nidulans* (Gems et al., 1991), vectors bearing this sequence were shown to self-replicate in species within the genera *Aspergillus*, *Penicillium*, *Giberella* (Aleksenko and Clutterbuck, 1997) as well as in *Trichoderma reseei* (Kubodera et al., 2002), *Lecanicillium* (Ishidoh et al., 2014), and *Paecilomyces variotii* (Seekles et al., 2021)*.* Telomeric sequences have also been reported to promote replication (and often integration) in various filamentous fungi like *A. nidulans* (Kistler and Benny, 1992), *Fusarium oxysporum* (Powell and Kistler, 1990), and *Chrysosporium lucknowense* (Verdoes et al., 2007). All of these vectors can be used efficiently for rapid assembly and delivery of transcription units expressing the gene(s) of interest into the host organism. The copy number of vectors maintained within the host differs by fungal species, and it is also influenced by the strength of the selection marker or the cultivation conditions. *Aspergillus* strains were shown to maintain numerous copies of AMA1 vectors in one nucleus (Fierro et al., 1996). Since they do not integrate in the genome, these vectors are easily lost without marker selection pressure (Aleksenko and Clutterbuck, 1997), which allows easy recycling of the same vector-based system.

In an impressive study, fungal artificial chromosomes based on AMA1 shuttle vectors have been used to capture the entire genome of *A. terreus* and to successfully clone all of its native BGCs in *A. nidulans,* resulting in the discovery of the astechrome biosynthetic machineries (Bok et al., 2015). The same approach was used to clone and overexpress 56 BGCs from other *Aspergillus* species in *A. nidulans*, resulting in the discovery of 15 novel metabolites (Clevenger et al., 2017). Although these shuttle vectors contained the BGCs with their native promoters, most of these compounds were not produced in the native hosts. Activation of these cryptic BGCs could be due to the presence of multiple copies of the vectors, or to the absence of native repressing factors such as epigenetic repression. Using fungal shuttle vectors in combination with modular cloning technologies and other well characterized advanced DNA assembly tools allows rapid refactoring and validation of multi-gene expression cassettes as well as synthetic metabolic pathways (Sarkari et al., 2017; Mózsik et al., 2021b).

### Biosynthetic Gene Cluster Expression Using Polycistronic mRNA

To allow simultaneous expression of multiple genes from one established fungal promoter, and to avoid tedious promoter replacement of all genes in a BGC, the cluster can be reconstructed using so-called “Stop-Carry On,” or ribosomal “skipping,” sequences between genes cloned in a sequential organization. Viral 2A peptides have been shown to promote ribosomal skipping during translation from polycistronic mRNA (Sharma et al., 2012). Since its discovery, this method has been widely applied in eukaryotes for multiple protein delivery from a single transcript. The 2A peptide sequences have been used to express the three genes of the penicillin BGC from one polycistronic mRNA in *A. nidulans* (Unkles et al., 2014). As the same promoter is driving the expression of all the genes of the BGC an equimolar production of each enzyme might be expected, which can lead to imbalances in the pathway and accumulation of toxic intermediates (Hoefgen et al., 2018). Some technical limitations with P2-based BGC expression are potential enzyme activity problems created by the remnants of the 2A peptide sequence at the C-termini of the proteins, validation that all genes from the transcript are effectively translated, and tedious vector construction time (Hoefgen et al., 2018). To solve the first issue, the additional amino acids can be removed introducing Tobacco Etch Virus (TEV) endopeptidase recognition sequences and co-expressing this peptidase in the host, and a seamless cloning step has been utilized to clone the genes of the BGC and label them with P2 and TEV recognition sequences (Hoefgen et al., 2018). To solve the second issue, it is possible to incorporate a split fluorescent reporter to ensure that the first and last genes are correctly translated. This advanced 2A-based expression system was applied for the heterologous expression of the austinoid BGC (∼13 kb) from *A. calidoustus* and the psilocybin BGC (∼7.4 kb) from *Psilocybe cubensis* in *A. nidulans* (Hoefgen et al., 2018).

### Filamentous Fungi as Platforms for the Heterologous Production of Secondary Metabolites

Because the great majority of fungi cannot be cultivated under laboratory conditions, model host strains are required for the heterologous expression of fungal BGCs and product identification. While common organisms as *E. coli* and *S. cerevisiae* have been successfully used for this purpose in some cases (Gao et al., 2010; Haynes et al., 2011; Davison et al., 2012; Bond et al., 2016; Zhang J. J. et al., 2019; Ma et al., 2021), filamentous fungi are much more suitable hosts for BGC expression for several reasons. Firstly introns do not strictly need to be removed when cloning a putative biosynthetic gene of interest, since filamentous fungi are more likely able to process them accurately during splicing, yielding the correct mRNA. Naturally, the chances of correct splicing are higher when cloning genes from organisms that are more closely related to the host of choice (Karnaukhova et al., 2007). The chances of a successful expression are further increased by a more ideal codon usage (Su et al., 2012). Secondly, fungi are more likely to produce the building blocks utilized by biosynthetic enzymes for secondary metabolite biosynthesis, because they are naturally wired for such processes. For the same reason, these hosts possess a plethora of accessory enzymes that are required for the correct functioning of the BGCs enzymes, such as phosphopantetheinyl transferases (PPTases), redox partners for P450s, prenyltransferases, and other enzymes (Keller, 2019). Additionally, hosts such as *A. niger*, *A. oryzae* are classified as GRAS (generally recognized as safe) organisms, and therefore they are suitable for the industrial production of compounds destined to be used in humans. The most commonly employed hosts for the characterization of heterologous BGCs are *A. nidulans* and *A. oryzae* (Anyaogu and Mortensen, 2015; Meng et al., 2021; Meyer, 2021), but other species have been successfully developed into platform strains, as showcased in Table 1. In the following section, we will discuss the most relevant examples and highlight their major features.

**TABLE 1:** Examples of fungal expression platforms for the production of natural products and the characterization of biosynthetic gene clusters.

Species	Platform strains	Genotype	Examples of NP	References
*Aspergillus nidulans*	A1145	pyrG89; *pyroA4; nkuA::argB; riboB2*	Flavunoidine	Yee et al. (2020)
	A1145 ΔSTΔEM	A1145 Δ*stc*-BGC, Δ*eas*-BGC	Myceliothermophin	Li et al. (2016)
	LO4389	A1145 Δ*stcA-stcW*	Zaragozic acid A precursor	Liu et al. (2017)
	LO8030	A1145 Δ*stc*-BGC, Δ*eas*-BGC, Δ*afo*-BGC, Δ*mdp*-BGC, Δ*tdi*-BGC, Δ*aus*-BGC, Δ*ors*-BGC, Δ*apt*-BGC	Trihazone A-F	Zhu et al. (2021)
	YM87 & YM137	LO4389 AN1029::*PalcA*-AN1029; AN1036-AN1032 (31)::*AfriboB*	Asperfuranone	Chiang et al. (2013)
		A1145 Δstc-BGC, Δeas-BGC	Aspercryptin	Chiang et al. (2016)
		A1145 ΔstcA-stcW	Felinone A	Oakley et al. (2017)
			Citreoviridin	Chiang et al. (2020)
			Mutilin	Chiang et al. (2020)
			Pleuromutilin	Chiang et al. (2020)
*Aspergillus oryzae*	NSAR1	*niaD−, sC−, ΔargB, adeA-*	Strobilurin	Nofiani et al. (2018)
	NSARΔK	NSAR1 Δ*kojA*	Paxilline	Tagami et al. (2013)
	NSPlD1	*niaD−, sC−, pyrG−, ligD-*	Erinacine Q	Liu et al. (2019a)
			Pretenellin A	Dao et al. (2021)
			Kojic acid	Yamada et al. (2014)
*Aspergillus niger*	AB1.13	*pyrG1, prtT-*	Enniatin	Richter et al. (2014)
			Beauvaricin	Boecker et al. (2018)
			Bassianolide	Boecker et al. (2018)
*Aspergillus terreus*	ΔakuB	SBUG844 Δ*akuB::hphR*	Isoflavipucine	Gressler et al. (2011)
	HZ03	Δ*ku80::ptrA, *Δ*pyrG*	Dihydroisoflavipucine	Gressler et al. (2011)
			Monacolin J	Huang et al. (2019)
*Penicillium rubens*	4xKO	∆*hdfA*, ∆*pen*-BGC, ∆*chy*-BGC, ∆*roq*-BGC: *amdS*, ∆*hcpA: ble*	Penicillin	Pohl et al. (2020)
			Decumbenone A-C	Pohl et al. (2020)
*Trichoderma reseei*	ΔpyrG	QM6a Δ*tmus53, *Δ*pyrG*	Sorbicillinoids	Derntl et al. (2017)

The filamentous fungus most widely used as heterologous host is by far *A. nidulans*. This species has been used for decades to study important cellular processes such as recombination, DNA repair, and chromatin regulation (Morris, 1976; Chaveroche, 2000; Osmani and Mirabito, 2004), because it can be easily manipulated and cultivated in the lab. This has led to the development of several platform strains that have been engineered to characterize and overexpress heterologous genes and, ultimately, produce natural products. The most interesting platform strains, showcased in Table 1, are derived from a triple auxotrophic strain called A1145 (Nayak et al., 2006). This strain also carries a deletion of the *nkuA* gene (homolog of human *ku70*) that renders NHEJ DNA repair less favorable, facilitating precise genomic integration of heterologous genes *via* HR. A1145 has been successfully used to elucidate a diverse range of biosynthetic pathways belonging to all major classes of SMs (Li et al., 2016; Hai and Tang, 2018; Liu L. et al., 2019; Yee et al., 2020). Despite these attractive features, *A. nidulans* also has a major downside. It is a prolific producer of SMs (Anyaogu and Mortensen, 2015; Meng et al., 2021) that typically yields a crowded chromatographic background, which can render the identification of new metabolites a cumbersome task. To partially overcome this problem, strains with a cleaner SM background have been developed. In one instance, the BGCs responsible for the biosynthesis of sterigmatocystin and emericellamids, two major classes of compounds produced by *A. nidulans*, were deleted. This allowed the identification of an important intermediate of the cholesterol-lowering compound zaragozic acid A in an engineered strain, previously overshadowed by the host metabolites (Liu et al., 2017). A similar strain—LO4389—where only the sterigmatocystin pathway was deleted, was used for the identification of 6 polyketides from *A. terreus* along with the complete reconstitution of the *A. terreus* asperfuranone pathway (Chiang et al., 2013), underlining once again the potential of such cleaner platform strains. Following the success of such trials, the same authors reported the construction of strain LO8030 where eight of the most highly expressed BGCs were deleted, resulting in a considerable reduction of the genome size (Chiang et al., 2016). Despite that, the strain showed no significant defects in growth. Additionally, not only did the strain offer a minimal background, but it also benefitted from a higher availability of SM precursors, as demonstrated by the synthesis and detection of the previously undiscovered metabolite aspercryptin (Chiang et al., 2016). Recently, LO4389 was used to construct two new strains that possess genetic features that are especially advantageous for the expression of entire biosynthetic pathways. In these strains, up to 6 or 7 genes of interest (GOIs) can be placed under the control of the native regulatory elements of the asperfuranone pathway, whose genes have been removed, while the inducible promoter P*alcA* controls the major TF regulating the BGC pathway. This elegant approach allows for the induction of entire heterologous BGCs upon the addition of methyl ethyl ketone, and its potential was demonstrated by the successful production of citreoviridin, mutilin, and pleuromutilin (Chiang et al., 2020).

Another common choice as a cell factory for the identification and production of SMs is *A. oryzae*. This fungus plays an important role in food manufacturing in Asia, where it is widely used for the production of alcoholic beverages and fermented products such as soy sauce and miso. *A. oryzae* contains numerous BGCs in its genome, many of which are also found in the toxins-producing species *A. flavus*, which is a prolific producer of natural products. In fact, the species are so closely related that it is believed that *A. oryzae* is actually a product of the domestication of A*. flavus* (Payne et al., 2006). However, *A. oryzae* produces only a few endogenous SMs, which makes it a perfect host for heterologous production (Anyaogu and Mortensen, 2015). The most common platform strain is the quadruple auxotroph NSAR1 (Jin et al., 2004) which offers great versatility and does not require the need of expensive additives for the selection of the transformants. NSAR1 has been used successfully by many researchers to elucidate diverse biosynthetic pathways (Tagami et al., 2013; Nofiani et al., 2018; Liu C. et al., 2019; Han et al., 2021). Despite the fact that its use is relatively more recent than *A. nidulans,* an extensive toolkit is now available to transform *A. oryzae* and express heterologous genes (Pahirulzaman et al., 2012; Lazarus et al., 2014). Of particular interest is a system of vectors—pTYargB/niaD/adeA/sC-eGFPac—that carry an inducible expression cassette (under control of the *amyB* promoter and terminator) and three constitutive cassettes. There are four versions of these vectors, each carrying a different selection marker, that can be co-transformed to allow the overexpression of up to 16 heterologous genes in the recipient NSAR1 strain (Lazarus et al., 2014). Although *A. oryzae* already offers a clean SM background, it produces a relatively abundant compound called kojic acid. The presence of kojic acid can complicate the purification of metabolites of interest and interfere with structural characterization. Deletion of the gene *kojA*, that encodes a crucial oxidoreductase for the synthesis of kojic acid, resulted in an astoundingly clean SM background and easy detection of the metabolite of interest (Dao et al., 2021). Another platform strain that merits attention is the triple auxotroph NSlPD1 (Maruyama and Kitamoto, 2008). The advantage of this particular strain over NSAR1 and its derivative strains is the deletion of the *ligD* gene, which facilitates HR-mediated genetic engineering (analogously to the deletion of *nkuA* in *A. nidulans*). NSlPD1 was successfully engineered to generate a strain that produces higher titers of kojic acid and uses cellulose as starting material (Yamada et al., 2014).

Two other well-known Aspergilli that have been explored as hosts for the biosynthesis of natural products are *A. niger* and *A. terreus*. The former has been used for the efficient production of the depsipeptides enniatin (Richter et al., 2014), beauvericin, and bassianolide (Boecker et al., 2018). These compounds show high insecticidal activity (Grove and Pople, 1980) and some have been proposed as potential candidates for the treatment of HIV infections (Shin et al., 2009). *A. terreus* is less commonly used as a heterologous host, but its tremendous natural capabilities as a producer of SMs (Huang et al., 2021) suggest that it could represent a worthy alternative to other fungal species, especially for the production of polyketides. In fact, this species is widely used for the industrial production of lovastatin (Boruta and Bizukojc, 2017), an essential pharmaceutical, that is, used to treat high blood cholesterol. In recent years, platform strains of *A. terreus* have been used to elucidate the biosynthetic pathway of the mycotoxin flavipucine (Gressler et al., 2011), and to generate a high-performance strain capable of producing high titers of monacolin J, a key precursor to the synthesis of semi-synthetic statins (Huang et al., 2019).

Another important fungal workhorse for industrial applications is *P. rubens*, which is used for the production of penicillin, cephalosporin, and other β-lactam antibiotics. To increase the titers of penicillin produced, early strains of *P. rubens* have been subjected to decades of random mutagenesis and selection processes, collectively known as the classical strain improvement program. This has also led to increased capabilities to grow in submerged cultivation conditions and in defined media, features that are very desirable in the industry (Harris et al., 2009; Guzmán-Chávez et al., 2018; Iacovelli et al., 2021a). The CSI program also led to a major reduction of the expression and/or mutational inactivation of non-penicillin BGCs. One of the industrial penicillin producer strains was recently engineered to abolish production of β-lactam antibiotics, with the assumption that this strain would retain its metabolic capabilities while offering a cleaner background. Indeed, this particular strain was used to heterologously express the compactin BGC from *P. citrinum*, together with an engineered cytochrome P450 from *Amycolatopsis orientalis*. The newly engineered strain was able to catalyze the final hydroxylation step of compactin to the cholesterol-lowering drug pravastatin, with an impressive yield of more than 6 g/L in pilot scale fermentations (McLean et al., 2015). This idea was further explored even more recently, when the same β-lactam-deficient strain was used as a template to generate a quadruple deletion strain in which alongside the penicillin BGC three major BGCs (chrysogine, fungisporin, and roquefortine) were removed, resulting in a considerably clean SM background, ideal for natural product production (Pohl et al., 2020). As proof of concept, the SM-deficient platform strain was used to reintroduce the penicillin BGC, resulting in restored production of the antibiotic, and to overexpress an endogenous PKS (PKS17), leading to the production of YWA1, a common precursor to fungal pigments. Additionally, the strain was used for the successful reconstitution of the calbistrin BGC from *Penicillium decumbens*, which resulted in the heterologous production of decumbenone A, B, and C (Pohl et al., 2020). This strain is also devoid of the *hdfA* gene (homolog of human *ku70*) and is therefore suitable for HR-mediated genetic engineering. These results highlight the *P. rubens* 4xKO strain as a valuable option for SM research.

Another filamentous fungus worth mentioning is *T. reesei*. For decades, this organism has been used in industry for its astonishing ability to produce cellulolytic enzymes such as cellulases and hemicellulases (Bischof et al., 2016), but it never attracted natural product researchers, probably due to the broad availability of other hosts and a lack of well-developed synthetic biology tools. Recently, a strain which carries deletions for the genes *tmus53* (*ligD* homolog) and *pyrG* was engineered (Steiger et al., 2011). Analogously to other fungal hosts, the *Δtmus53* deletion facilitates HR-mediated targeted gene integration, while *ΔpyrG* allows easy selection through complementation of uracil/uridine auxotrophy. These features raise interest for *T. reesei* as a potential natural product producing host. In fact, this particular strain has already been utilized to investigate the endogenous biosynthetic pathway of sorbicillinoids (Derntl et al., 2017). What could set this platform apart and make it a concrete option for industrial applications is its natural ability to degrade and thrive on cellulosic material, which could lead to the production of natural products starting from biomass material, such as agricultural waste.

Undoubtedly, the fungal platforms discussed above provide a rich choice for researchers who want to investigate unknown biosynthetic pathways or produce industrially relevant metabolites. Nevertheless, despite the broad availability of hosts, not all species might be capable to produce the desired natural product, and even when they are, it is very likely that one species performs better than another in terms of yield. This is difficult to predict and engineering more species at once to optimize production can be extremely time-consuming as well as costly. To reduce the workload and facilitate simultaneous cloning and screening of more hosts, a group of researchers recently developed the first multispecies fungal platform for heterologous gene expression (Jarczynska et al., 2020). The first version of this system, called DIVERSIFY, is based upon four *Aspergillus* species: *A. nidulans*, *A. oryzae*, *A. niger*, and *A. aculeatus.* Each individual species was first engineered to contain in the genome a “common synthetic gene integration site” (COSI), which encodes a reporter gene for white/blue selection placed under the control of a constitutive promoter. Additionally, the COSI contains two 500 bp sequences flanking the reporter cassette that can be used for HR-mediated target gene replacement, whereby GOIs can be easily inserted and overexpressed. Since the COSI is equal in each recipient strain, only one integration cassette has to be designed and built. As a proof of concept, the DIVERSIFY platform has been successfully used to overexpress a fluorescent reporter (mRFP) and cellobiohydrolase, and for the production of 6-MSA, a model polyketide (Jarczynska et al., 2020). Because many of the synthetic biology tools developed for fungi are readily adaptable to other species, this platform can be expanded with other hosts in the future.

Clearly, filamentous fungi are powerful instruments for the elucidation of biosynthetic pathways and the production of SMs. In many cases, though, the desired compounds are produced at very low yields which are unsuitable for commercial applications. The development of a great array of hosts, each with specific benefits, as well as multispecies platforms that allow fast and simultaneous screening of several fungi with reduced workloads, will offer researchers the tools to readily optimize the production yield of metabolites of interest. Ultimately, this is necessary to engineer efficient fungal cell factories that are ready to be employed at an industrial scale.

## Concluding Remarks

**FIGURE 2:**
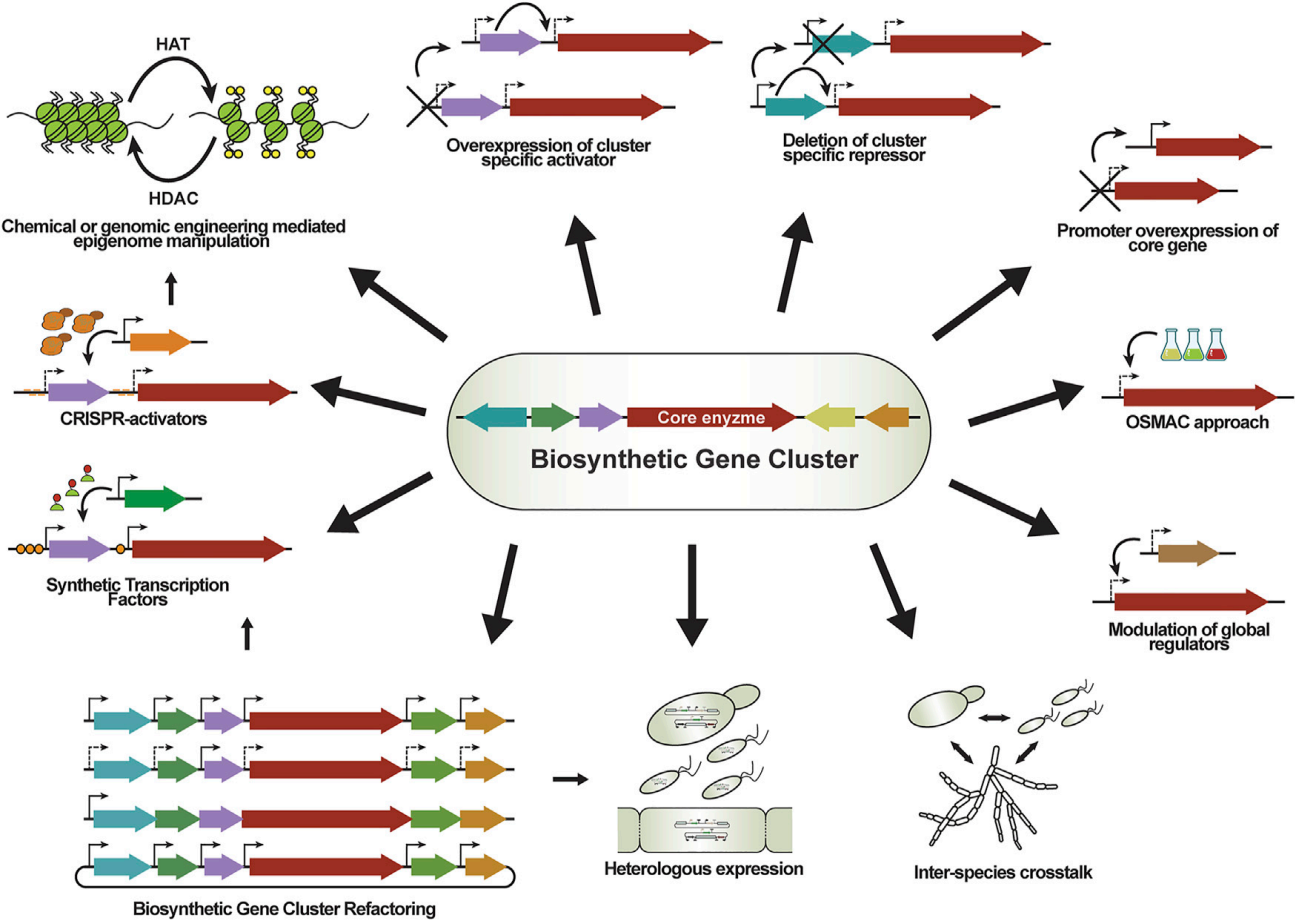
Strategies for transcriptional activation for fungal biosynthetic gene clusters. Dashed arrows indicate native, solid arrows indicate engineered (strong or inducible) promoters.

Thanks to the rapidly expanding number of filamentous fungal genome sequences and the advanced bioinformatics tools that are now available, it has become obvious that filamentous fungi represent an untapped reservoir of natural products. Each genome contains a high number of BGCs for which the product has not been identified, and many of these BGCs are transcriptionally silent under laboratory conditions. The products of these clusters can be unearthed with the combined efforts of bioinformatics, chemistry, and synthetic biology, to reveal new chemistries and biological activities of interest. A major challenge, however, remains prioritizing these BGCs for their potential value since the bioinformatics tools available at the moment cannot reliably predict the resulting products. Hence, much relies on laborious empirical testing, whereby many BGCs have to be expressed to screen for bioactive compounds. Here, we have discussed conventional tools and the development of new synthetic biology tools that aid in the transcriptional activation of silent BGCs in filamentous fungi, therefore offering new approaches for compound discovery (Table 2 and Figure 2).

**TABLE 2:** Transcriptional activation tools and methods for fungal biosynthetic gene clusters.

Transcriptional activation method	Benefits	Drawbacks
Overexpression of BGC core gene	• Reliable transcriptional activation of the targeted gene	• Although transcription is activated, product formation is not ensured
	• Limited genomic modulation needed	• Does not activate the complete BGC
Modulation of BGC-specific TF	• Limited genomic modulation needed	• Often no cluster-specific TFs are present in a BGC
	• Overexpression of positive regulator can upregulate entire BGCs	• Overexpression of such TF does not guarantee transcriptional activation of the entire BGC
		• Other co-activators, mediators or inducers might be needed for activation
Modulation of global regulators	• Limited genomic modulation needed	• Regulator needs to be identified
	• Multiple BGCs are affected, resulting in higher chances for compound discovery	• Global regulator targets are often unknown
		• Modulation can be lethal
		• Difficult to assign newly produced compounds to specific BGCs
Epigenome modulation	• Feeding of chemical modulators is easy to carry out	• Histone modifying enzymes have to be identified and engineered
	• Multiple BGCs are affected, resulting in higher chances for compound discovery	• Modulation can be lethal
		• Difficult to assign newly produced compounds to specific BGCs
BGC refactoring	• Native regulatory system is bypassed	• Requires extensive DNA cloning and/or synthesis efforts
	• Episomal delivery of BGCs can lift the burden of epigenetic repression	• Limited number of established promoters
	• Transcription relies solely on established promoters	
	• Fungal SM deficient strains are available	
Heterologous expression in non-fungal host	• Established heterologous systems and regulation tools are broadly available	• Potential problems with codon usage, available precursors, cellular trafficking, RNA splicing and post-translational modifications
STF-based BGC regulation	• Native regulatory system is eliminated or bypassed	• Extensive DNA cloning and/or DNA synthesis effort required
	• Transcription relies on an orthogonal regulatory system	• Genome editing or BGC refactoring is required
	• Modular features and scalable transcriptional regulation possible	• Validation (specificity, activity) of new STFs is necessary
CRISPR-based BGC regulation	• Genome editing-free transcriptional activation or repression	• Extensive DNA cloning and/or DNA synthesis effort required
	• Rapid library construction	• Genome editing or BGC refactoring is required
	• Various regulatory domains are available for transcriptional activation, repression or epigenetic modulation	• No established rules available for creating STF fusions
		• Preceding validation required (activity, specificity)

Modulating global regulatory systems does not require prior knowledge regarding the cluster-specific regulation mechanism of a given BGCs, and although numerous genes can be affected when using this approach, transcriptional activation of BGCs of interest and subsequent production of new metabolites are not guaranteed. Instead, overriding the native regulatory system of the cluster can result in a more direct transcriptional activation. Such approaches involve partial or complete BGC refactoring, and require a deep level of understanding about the number of genes in the BGC, and/or its cluster specific regulators. Refactoring approaches can take place within a host which supports the assembly of numerous DNA fragments, often achieved *via* homologous recombination or advanced synthetic cloning methods. The only approach that ensures transcriptional activation of an entire BGC is promoter replacement of all its genes, but this is generally a laborious and cumbersome task. Furthermore, the number of established strong fungal promoters and fungal selection markers is still limited, and this represents a bottleneck towards rapid or consecutive genomic modifications. An alternative to serial replacement of promoters could be the utilization of promoters from BGCs which are transcriptionally active in the selected host, or synthetic promoters containing regulatory elements for orthogonal regulatory systems such as STFs. The more recent CRISPRa systems further increase the number of available activator tools adding a new layer of control, since such systems do not require BGC refactoring anymore.

Since genetic manipulation and precise engineering of a non-model or wild type fungal strains is challenging—mainly due to the low rate of HR and difficulties to grow these organisms in laboratory conditions—the most versatile method is to express BGCs in an established heterologous host. Ideal host strains are those in which it is easy to perform genetic manipulation, that are convenient to cultivate at different scales, for which compatible genetic tools are available, and convenient downstream processing steps have been established such as rapid compound screening and a clean metabolite spectrum. A combination of advanced transcriptional activation tools and established expression hosts can ensure reliable, targeted transcriptional activation and efficient methods for compound identification.

Although new host strains and tools are continuously being developed for the characterization of cryptic BGCs in filamentous fungi, high-throughput BGC screening remains a major challenge. Automatized engineering of protoplasted filamentous fungi using microtiter plates and robotic liquid handling robots have been successfully established (Kuivanen et al., 2019), as well as fully-automated microscale bioreactor cultivations (Jansen et al., 2021), but working with BGC-coding DNA is demanding. The large size, the numerous genes, and the costs for total cluster DNA synthesis are limiting factors for rapid assembly and screening of numerous BGCs.

In the future, the combination of emerging genetic tools, tailored heterologous hosts with high metabolic capacity, and automated systems, will facilitate the development of highly efficient, targeted, multiplexing-compatible transcriptional activation applications for novel natural product discovery. This, coupled with the development of bioinformatics tools that are able to prioritize the most valuable BGCs within genomic sequences, will revolutionize the field and eliminate time-consuming and costly wet lab procedures, while starkly increasing the chances of identifying novel and potent bioactive compounds.

## Abbrevations

AD, activation domain; CP, core promoter; DBD, DNA-binding domain; STF, synthetic transcription factor; UAS, upstream activating sequence
